# Prevalence of Papillomaviruses, Polyomaviruses, and Herpesviruses in Triple-Negative and Inflammatory Breast Tumors from Algeria Compared with Other Types of Breast Cancer Tumors

**DOI:** 10.1371/journal.pone.0114559

**Published:** 2014-12-05

**Authors:** Marilys Corbex, Sabiha Bouzbid, Alexandra Traverse-Glehen, Hayette Aouras, Sandrine McKay-Chopin, Christine Carreira, Abdelaziz Lankar, Massimo Tommasino, Tarik Gheit

**Affiliations:** 1 Université Libre de Bruxelles, Brussels, Belgium; 2 Badji Mokhtar University, Faculty of Medicine, Annaba, Algeria; 3 University Hospital, Annaba, Algeria; 4 Hospices Civils de Lyon, Laboratoire d'Anatomie Pathologique, Université Lyon 1, Lyon, France; 5 Infections and Cancer Biology Group, International Agency for Research on Cancer, Lyon, France; 6 Molecular Pathology Group, International Agency for Research on Cancer, Lyon, France; 7 Cytology and pathology laboratory, University Hospital, Annaba, Algeria; University of Chile, Chile

## Abstract

**Background:**

The possible role of viruses in breast cancer etiology remains an unresolved question. We hypothesized that if some viruses are involved, it may be in a subgroup of breast cancers only. Epidemiological arguments drove our interest in breast cancer subgroups that are more frequent in Africa, namely inflammatory breast cancer (IBC) and triple-negative breast cancer. We tested whether viral prevalence was significantly higher in these subgroups.

**Materials and Methods:**

One hundred fifty-five paraffin-embedded malignant breast tumors were randomly selected at the pathology laboratory of the University Hospital of Annaba (Algeria) to include one third of IBC and two thirds of non-IBC. They were tested for the presence of DNA from 61 viral agents (46 human papillomaviruses, 10 polyomaviruses, and 5 herpesviruses) using type-specific multiplex genotyping assays, which combine multiplex PCR and bead-based Luminex technology.

**Results:**

Viral DNA was found in 22 (17.9%) of 123 tumors. The most prevalent viruses were EBV1 and HPV16. IBC tumors carried significantly more viruses (any type) than non-IBC tumors (30% vs. 13%, p<0.04). Similarly, triple-negative tumors displayed higher virus-positivity than non-triple-negative tumors (44% vs. 14%, p<0.009).

**Conclusions:**

Our results suggest an association between the presence of viral DNA and aggressive breast cancer phenotypes (IBC, triple-negative). While preliminary, they underline the importance of focusing on subgroups when studying viral etiology in breast cancer. Further studies on viruses in breast cancer should be conducted in much larger samples to confirm these initial findings.

## Introduction

Breast cancer (BC) is the second most common cancer worldwide, with 1.67 million new cases per year in 2012 [1]. It is the most common cause of death from cancer among women in less developed regions (324 000 deaths annually). Several risk factors have been identified, including sex, age, dense breast tissue, susceptibility genes, family history, ethnicity, hormones, and alcohol consumption. However, for most cases the initiating cause remains unexplained [2].

The International Agency for Research on Cancer (IARC) estimated that 15-20% of cancers are associated with infectious agents [3]. The discovery in 1944 that a virus caused mammary cancer in mice inspired scientists to explore a possible viral etiology in BC [4]. However, for decades, the results remained unclear and inconclusive, generating considerable controversies [5]. Recently, with improvements in techniques and some encouraging results, there has been a resurgence of interest in the possibility that a significant proportion of human BCs may be caused by viral infections [6]–[8]. The vast majority of studies conducted so far have focused on three viruses: mouse mammary tumor virus-like sequences (MMTV-LS) [9], [10], Epstein-Barr virus (EBV) [11]–[13], and human papillomavirus (HPV) [14]–[16], giving substantial but no conclusive evidence of a viral role in breast carcinogenesis. A recent systematic review focusing on these three viruses concluded that the evidence available to date remains preliminary and advocated for key improvements in methodological approaches, notably the use of appropriate epidemiological design to determine if the presence of viruses is significantly associated with some subgroup of BC (comparison across well-defined subgroups of cases) [7]. The present work builds on this recommendation and explores whether viruses are associated with higher risk of some well-defined subtypes of BC. The mixed results displayed in the literature so far are compatible with the idea that some viruses could act as co-factors in the oncogenic process and increase the risk of only some subtypes of BC.

We based the present work on the hypothesis that if a virus that increases risk of BC exists, it may account for a higher attributable fraction of BC in developing countries than in industrialized countries, where almost all studies of viruses and BC have been conducted to date. Indeed, it has been shown that the proportion of cancers caused by viruses is substantially higher in developing countries, notably in Africa: in Western Europe, it is estimated to be 7% of all cancer cases, whereas this proportion reaches 40% on the African continent [3]. We further hypothesized that the putative virus may be found preferentially in some subgroups of BC that are relatively more frequent in Africa than in Western countries.

Inflammatory BC (IBC), a particularly aggressive form of BC [17], is a subgroup of interest that is rare in developed countries, where it represents 2% to 3% of all cancers, whereas in North Africa its proportion is significantly higher, reaching 10% [18], [19] .

Triple-negative BC, defined as BC that is negative for estrogen receptor (ER), progesterone receptor (PR), and human epidermal growth factor receptor 2 (HER2), is another subgroup of interest since recent observations have suggested that this subtype of BC is more frequent in less developed countries (17% to 28%), notably in Africa [19]–[24], compared with Europe and North America, where the proportion of triple-negative BC is about 10–12% [25], [26].

The relation between IBC and triple-negative status has not been much studied, but the few studies published to date suggest that IBC may be triple-negative more frequently (20% to 40% of cases) than non-IBC [27]–[29].

The present study was conducted on samples from Algeria, a North African country. The two main objectives of the study were (1) to compare viral prevalence in IBC and non-IBC, and (2) to compare viral prevalence in triple-negative and non-triple-negative BC tumors. Viral prevalence was tested for using highly sensitive bead-based multiplex genotyping assays for the detection of 46 mucosal and cutaneous human papillomavirus types, 10 polyomaviruses including the simian polyomavirus SV40, and 5 herpesviruses.

## Materials and Methods

### Samples

Tumors from patients of Algerian ethnic origin diagnosed between 2008 and 2011 at the University Hospital of Annaba were considered. The use of this tumor collection for research purposes was approved by the ethics committee of the University Hospital of Annaba. The ethics committee waived the need for written informed consent for the present study. Fifty IBC tumors and 105 non-IBC tumors, fixed in paraffin, were selected randomly from the cytology and pathology laboratory database. Epidemiological and demographic data were extracted retrospectively from medical records and cancer registry records and entered in EpiData software. The following were documented: morphology, tumor–node–metastasis (TNM) stage, “Poussée Evolutive” (PEV) stage (a clinical classification developed at Institut Gustave Roussy in the 1960s that is based on the rate of development of the tumor and the extent of the involvement of the breast rather than the tumor size), hormone receptor subtypes (ER/PR/HER2), age, housing type, socio-economic level, and menopausal status. Socio-economic level (high, medium, or low) was inferred from the district where the patient lived and her health insurance scheme. Since subtype testing is a recent technological advance, information about ER/PR/HER2 subtypes was not available for all patients, and notably was missing for those diagnosed before 2009. Inflammatory status was mentioned in the medical records, but patients were included as IBC in the final sample of the present study only if the IBC clinical diagnosis was corroborated by TNM (T4d) or PEV status (PEV3). Patients who were included as non-IBC in the final sample were all PEV0 (one tumor was excluded because of lack of information on the PEV status), and none were T4d. The final study sample included 45 confirmed IBCs and 104 confirmed non-IBCs. ER/PR/HER2 subtypes were available for 96 of these 149 patients.

### Preparation of the Paraffin Sections and DNA Extraction

All paraffin blocks were processed at IARC, Lyon. Three sections of 10 µm were cut from each paraffin block. For each specimen, the blades were changed and the microtome extensively washed with DNA Away (Dutscher, Brumath, France) to prevent the risk of cross-contamination between different specimens during the cutting. In addition, empty paraffin blocks were cut every 10 tumor specimens and blindly analyzed to monitor possible cross-contamination. No contamination was detected throughout the study.

DNA was prepared by incubating the paraffin tissue sections in digestion buffer (10 mM Tris/HCl [pH 7.4], 0.5 mg/ml proteinase K, and 0.4% Tween 20) overnight at 37°C. Samples were incubated for 10 min at 95°C, centrifuged rapidly for 2 min at 15700*g* in a bench centrifuge, and chilled on ice, to inactivate the proteinase K and to remove the paraffin. Finally, the aqueous phase was transferred to a new tube.

### Detection of Viral DNA

The identification of 61 infectious agents was performed by using type-specific multiplex genotyping (TS-MPG) assays, which combine multiplex polymerase chain reaction (PCR) and bead-based Luminex technology (Luminex Corp., Austin, TX, USA), as described previously [30]–[33]. Multiplex type-specific PCR uses specific primers for the detection of 19 probable/high-risk alpha-HPV types (HPV 16, 18, 26, 31, 33, 35, 39, 45, 51, 52, 53, 56, 58, 59, 66, 68a, 68b, 70, 73, and 82), 2 low-risk alpha-HPV types (HPV 6, 11), 25 genus-beta HPV types (HPV 5, 8, 9, 12, 14, 15, 17, 19, 20, 21, 22, 23, 24, 25, 36, 37, 38, 47, 49, 75, 76, 80, 92, 93, and 96), 10 polyomaviruses (BKV, KIV, JCV, MCV, WUV, TSV, HPyV6, HPyV7, HPyV9, and SV40), and 5 herpesviruses (CMV, EBV1, EBV2, HSV1, and HSV2). Two primers for the amplification of beta-globin were also added to provide a positive control for the quality of the template DNA [34].

### In situ Hybridization for Epstein-Barr encoding region (EBER)

In situ hybridization for EBER was performed on EBV DNA positive cases using a FITC-labeled oligonucleotide probe (Ventana Medical Systems, Tucson, AZ, USA) on an automated stainer (Ventana BenchMark). Visualization was achieved using the ISH iView system with Alk-Phosphatase and NT/BCIP substrate, counterstained with red (Counterstain II; Roche Diagnostic GmbH, Mannheim, Germany).

### Statistical Analyses

Statistical analyses were performed using SAS software (SAS Institute, Cary, NC, USA). For most analyses, the Fisher exact Chi-square test, which enables the testing of very small numbers, was used. When tables were larger than 2×2, the classical Pearson Chi-square test was used.

## Results

The demographic and clinical data for IBC and non-IBC patients are summarized in **Table 1**. The two groups of patients did not differ significantly in age, socio-economic variables (housing type, education level, socio-economic level), or reproductive variables (menopausal status, number of children, breastfeeding duration). However, they did differ significantly in ER/PR/HER2 status: 50% of the IBC patients for whom ER/PR/HER2 subtypes were available were triple-negative, whereas only 23% of such non-IBC patients were triple-negative (see **Table 1**).

**Table 1:** Profile of the IBC and non-IBC patients.

Characteristic	IBC	Non-IBC	*Comparison*
	N = 45	N = 104	*p-value*
**Age** (years, mean)	48.1	48.8	*0.70*
**Housing type**			
Urban	22	65	
Rural	23	38	*0.14*
**Education level**			
None	18	36	
Primary	7	21	
Secondary	10	19	
Tertiary/University	9	25	*0.78*
**Socio-economic level**			
Low	24	46	
Medium	19	45	
High	2	11	*0.39*
**Menopausal status**			
Yes	19	50	
No	26	54	*0.59*
**Number of children** (mean)	3.79	3.35	*0.30*
**Breastfeeding duration**			
None	8	22	
≤1 year	10	25	
>1 year	26	57	*0.88*
**ER/PR/HER2 status**			
ER+ or PR+ or HER2+	13	54	
Triple-negative	13	16	*0.013*

IBC, inflammatory breast cancer.

The amplification of the beta-globin gene showed that good-quality DNA could be obtained for 123 (82.6%) of the 149 paraffin-embedded tissue samples. The 26 samples negative for the beta-globin test were thus excluded from the analyses.

The presence of viral DNA was detected in 22 (17.9%) of the 123 tumors. As detailed in **Table 2**, most of the viruses tested for were absent from the tumors. The most prevalent virus was EBV1 (found in 10 tumors), followed by HPV16 (in 8 tumors), HPV31 (in 3 tumors), and HPV22 (in 2 tumors). HPV5, HPV6, and Merkel cell polyomavirus (MCV) were found in one tumor each. Four tumors displayed co-infection, but this number was not significantly above what would be expected by chance (p = 0.07); however, our statistical power to detect something significant at this level was very low. Of the four co-infections, two were non-IBC and were positive for EBV and HPV16; the other two were IBC, and one was positive for HPV31 and EBV and the other for HPV31 and MCV.

**Table 2:** Presence/absence of viral DNA in 123 breast cancer tumors.

Virus	Positive tumors	Virus	Positive tumors	Virus	Positive tumors
HPV5	1				
HPV8	0	HPV6	1		
HPV9	0	HPV11	0	BKV	0
HPV12	0	HPV16	8	KIV	0
HPV14	0	HPV18	0	JCV	0
HPV15	0	HPV26	0	MCV	1
HPV17	0	HPV31	3	WUV	0
HPV19	0	HPV33	0	TSV	0
HPV20	0	HPV35	0	HPyV6	0
HPV21	0	HPV39	0	HPyV7	0
HPV22	2	HPV45	0	HPyV9	0
HPV23	0	HPV51	0	SV40	0
HPV24	0	HPV52	0		
HPV25	0	HPV53	0	**Total polyomaviruses**	1
HPV36	0	HPV56	0		
HPV37	0	HPV58	0		
HPV38	0	HPV59	0		
HPV47	0	HPV66	0	CMV	0
HPV49	0	HPV68a	0	EBV1	10
HPV75	0	HPV68b	0	EBV2	0
HPV76	0	HPV70	0	HSV1	0
HPV80	0	HPV73	0	HSV2	0
HPV92	0	HPV82	0		
HPV93	0				
HPV96	0				
**Total beta-HPV**	3	**Total alpha-HPV**	12	**Total herpesviruses**	10

The presence of viral DNA according to IBC status is detailed in **Table 3**. There were significantly more viruses of any type in IBC tumors (30% virus-positive) than in non-IBC tumors (13% virus-positive) (p<0.04), but no unique type of virus was found significantly more frequently in IBC tumors than non-IBC tumors.

**Table 3:** Presence/absence of viral DNA according to IBC status.

Virus	IBC	Non-IBC	OR [95% CI]
	N = 37 N (%)	N = 86 N (%)	*p-value* *
**Any virus** **			
Absent	26 (70%)	75 (87%)	2.9 [1.1–7.4]
Present	11 (30%)	11 (13%)	*0.038*
**EBV1**			
Absent	32 (86%)	81 (94%)	2.5 [0.6–9.4]
Present	5 (14%)	5 (6%)	*0.16*
**HPV16**			
Absent	34 (92%)	81 (94%)	1.4 [0.3–6.3]
Present	3 (8%)	5 (6%)	*0.69*
**HPV31**			
Absent	35 (95%)	85 (99%)	4.8 [0.4–55]
Present	2 (5%)	1 (1%)	*0.21*
**HPV22**			
Absent	36 (97%)	85 (99%)	2.3 [0.1–39]
Present	1 (3%)	1 (1%)	*0.51*
**MCV**			
Absent	36 (97%)	86 (100%)	*-*
Present	1 (3%)	0 (0%)	*0.30*

*Fisher exact chi-square test

**Presence of DNA of any of the following viruses: BKV, KIV, JCV, MCV, WUV, TSV, HPyV6, HPyV7, HPyV9, SV40, CMV, EBV1, EBV2, HSV1, HSV2 and 46 types of HPV (21 alpha-HPV and 25 beta-HPV)

The presence of viral DNA according to ER/PR/HER2 status is detailed in **Table 4**. There were significantly more viruses of any type in triple-negative tumors (44% virus-positive) than in non-triple-negative tumors (14% virus-positive) (p<0.009). EBV was significantly more frequent in triple-negative tumors (24% EBV-positive) than in non-triple–negative tumors (2% EBV-positive) (p<0.003). No other virus differed significantly in frequency between triple-negative and non-triple-negative tumors.

**Table 4:** Presence/absence of viral DNA according to ER/PR/HER2 status.

Virus	Triple-negative	ER+ or PR+ or HER2+	OR [95%CI]
	N = 25 N (%)	N = 56 N (%)	*p-value* *
**Any virus** **			
Absent	14 (56%)	48 (86%)	4.7 [1.6–14]
Present	11 (44%)	8 (14%)	*0.0088*
**EBV1**			
Absent	19 (76%)	55 (98%)	17.3 [1.9–153]
Present	6 (24%)	1 (2%)	*0.0029*
**HPV16**			
Absent	21 (84%)	53 (%)	3.3 [0.7–16]
Present	4 (16%)	3 (%)	*0.19*
**HPV31**			
Absent	24 (96%)	55 (98%)	2.2 [0.1–38]
Present	1 (4%)	1 (2%)	*0.52*
**HPV22**			
Absent	24 (96%)	55 (98%)	2.2 [0.1–38]
Present	1 (4%)	1 (2%)	*0.52*
**MCV**			
Absent	24 (96%)	56 (100%)	*-*
Present	1 (4%)	0 (0%)	*0.31*

*Fisher exact chi-square test

**Presence of DNA of any of the following viruses: BKV, KIV, JCV, MCV, WUV, TSV, HPyV6, HPyV7, HPyV9, SV40, CMV, EBV1, EBV2, HSV1, HSV2 and 46 types of HPV (21 alpha-HPV and 25 beta-HPV)

In order to rule out potential confounding factors, we investigated the viral prevalence (for all viruses, EBV, and HPV16) by age, housing type, education level, and socio-economic level. We found no significant difference in viral prevalence according to these variables, confirming that these variables could not be confounding factors (data not shown).

As IBC correlated with triple-negative subtype (see **Table 1**), we tested whether the association between IBC status and presence of virus (any type) holds after adjustment for triple-negative status. The association was found to hold in non-triple-negative tumors (odds ratio [OR] = 7.0, *p*<0.03) but not in triple-negative tumors (see **Table 5**).

**Table 5:** Presence/absence of viral DNA in IBC/non-IBC tumors, stratified by triple-negative status.

	Triple-negative		Non-triple-negative	
Any virus	IBC	Non-IBC	IBC	Non-IBC
Absent	6	8	6	42
Present	6	5	4	4
	OR = 1.6		OR = 7.0	
	p = 0.69*		p = 0.029*	

*Fisher exact chi-square test

In our sample, IBC correlates with triple-negative subtypes, indicating that these two aggressive forms of BC may have some common risk factors. Therefore, we also compared the presence of viral DNA in the subgroup of aggressive tumors, defined as IBC or triple-negative (N = 50), with that in the subgroup of non-aggressive tumors, defined as non-IBC or non-triple-negative (ER+ or PR+ or HER+) (N = 46). The results were quite similar to those obtained in Table 4 with triple-negative tumors, i.e. significant differences for EBV and the “any virus” group (data not shown). Viruses were present in 32% of aggressive tumors compared with 9% of non-aggressive tumors (p<0.006).

**Figure 1:**
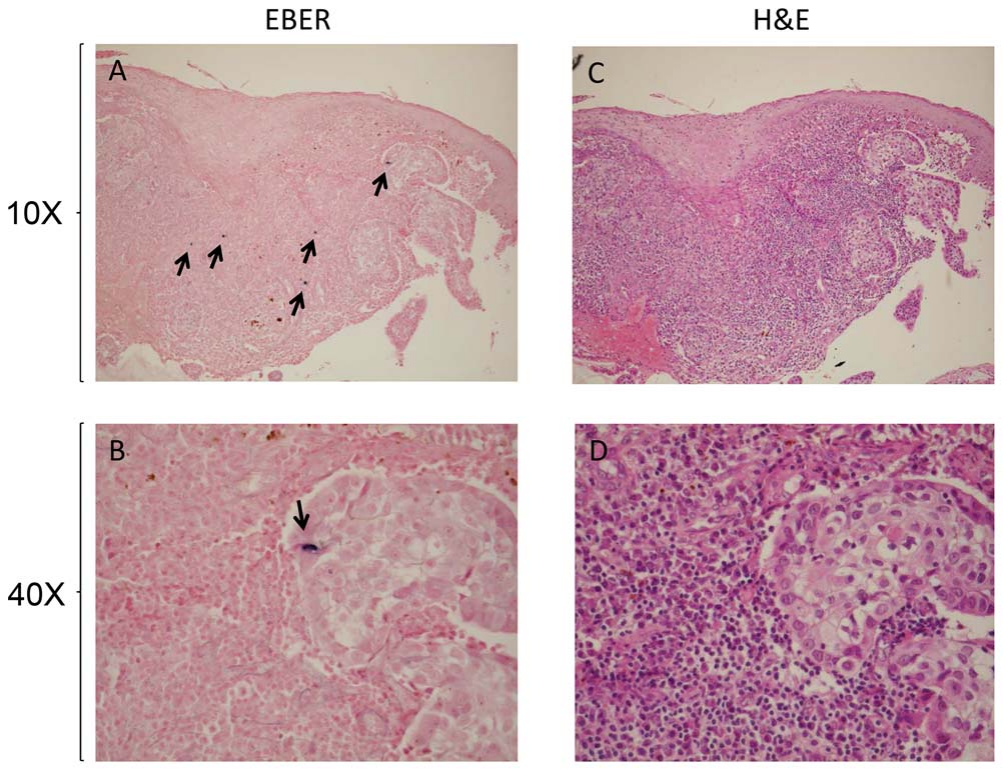
In situ hybridization for Epstein-Barr encoding region (EBER) in breast carcinoma shows (A) a few malignant epithelial cells and lymphocytes that express EBER (magnification x10) and (B) one malignant mammary epithelial cell with strong nuclear labeling indicating EBER expression (magnification x40). (C and D) Hematoxylin and eosin (H&E) staining performed on the same breast carcinoma (magnifications x10 and x40).

In situ hybridization for EBER was performed on 10 EBV DNA positive cases in order to determine whether EBV expression occurs in tumor epithelial cells or in infiltrating lymphocytes. Only 1 out of 10 cases, an IBC co-infected with EBV and HPV31, showed rare mammary tumor epithelial cells with nuclear staining for EBER (Figure 1B). EBV-infected lymphocytes were also detected in the stroma of that case (Figure 1A).

## Discussion

The major finding of the present study is that IBC and triple-negative BC have a higher tendency to be positive for oncogenic viruses, mainly EBV and HPV. Our results suggest that these viruses could act as co-factors in the oncogenic process that lead to a particularly aggressive form of BC. The study has several limitations, including the relatively small sample size and the absence of control (healthy breast tissue), but it also has some unique strengths compared with previous studies; these are (1) the very large number of viruses tested for (to date no study has explored the presence of so many oncogenic viruses in BC), (2) the high quality of the laboratory assays, and (3) the rigorous collection and use of critical epidemiological information.

Overall, we found a relatively low prevalence of viral DNA in our tumors from Algerian patients (18% of tumors were virus-positive). This is not attributable to the quality of the samples, because the amplification of the beta-globin gene ensured that all samples included in the analyses contained DNA of good quality. The low number of positive samples did not confer high statistical power to observe differences between subgroups; however, we found significant differences among the two subgroups we had chosen to study based on epidemiological arguments. There were significantly more viruses in IBC tumors (30% virus-positive) than in non-IBC tumors (13% virus-positive) (p<0.04), and there were significantly more viruses in triple-negative tumors (44% virus-positive) than in non-triple-negative tumors (14% virus-positive) (p<0.009). IBC and triple-negative status correlated in our sample (50% of IBC tumors were triple-negative vs. 23% of non-IBC tumors, p<0.02); the subgroup of aggressive tumors, defined as IBC or triple-negative, contained viral DNA in 32% of cases, compared with 9% for the subgroup of non-aggressive tumors (p<0.006).

EBV was the most common virus. It was significantly more prevalent in triple-negative tumors (24%) than in non-triple-negative tumors (2%) (p<0.003) and also appeared more prevalent in IBC tumors (14%) than in non-IBC tumors (6%), although this difference was not significant. Studies in larger samples are required to confirm these associations.

Both IBC and triple-negative cancers are known to be more aggressive than the other types of BC [17],[20]. Both are slightly associated with young age and with low socio-economic status [17], [23]. So we investigated whether age or socio-economic variables were confounding factors in the association described above. The prevalence of viruses did not differ according to age or socio-economic variables, indicating that these factors are not involved in the difference we observed between the BC subgroups of interest.

To date, only three studies have explored the prevalence of viral infection in IBC. In 2001, Fina et al. compared EBV load in 22 IBC and 76 non-IBC frozen tumors from Algeria and Tunisia and found no significant difference. The prevalence of EBV in their samples (40% of IBCs EBV-positive) was much higher than in ours [35]. In 2009, Pogo et al. found MMTV-LS in 44 (72%) of 61 paraffin-embedded IBC tumors, but did not compare with non-IBC tumors [36]. Recently, El Shinawi et al. searched for MCV sequences in DNA extracted from 28 IBC and 48 non-IBC fresh tumors from Egypt and found that IBC tumors were significantly more positive for MCV DNA compared with non-IBC tumors (78% vs. 53%) [37].

To our knowledge there is no large study exploring viral infection in triple-negative BC, but some studies exploring association with hormone receptor subtypes have been conducted. In a well-conducted study of 196 frozen tumors from the south of France, Mazouni et al. found that EBV positivity was significantly more frequent among ER- than ER+ samples (45.4% vs. 27.9%) [38], which is consistent with our results. In a study of 50 frozen breast tumors, Glenn et al. found higher prevalence of EBV, HPV, and MMTV among young cases and high-grade tumors but no variation according to ER, PR, or HER2 status; however, the statistical power to observe such a variation was low [12]. In a study of 85 paraffin-embedded BC tumors from Brazil, Ribeiro-Silva et al. found no correlation between ER or PR status and EBV EBNA-1 protein expression, but their statistical power to do so was limited [39]. A few other studies of viral prevalence that take ER status into account have been conducted, but they suffer from very small sample sizes [40] or methodological flaws, which make them poorly informative.

Our results on EBV corroborate those of the about 30 studies that report the presence of EBV in BC tumors (for reviews, see [12] and [7]). EBV is a co-factor in the development of various malignancies, including several types of carcinomas (nasopharyngeal carcinomas, gastric carcinomas) [41]. It is now well established that EBV can be found in BC tumors, thanks to specific PCR techniques [12]. A frequent critique in the past was that most of the PCR techniques applied to whole tumors could not distinguish between cancer cells and infiltrating lymphocytes. However, in a definitive study of 509 tumors, using in situ hybridization and laser capture microdissection combined with quantitative PCR, Fina et al. showed that EBV localization was restricted in the breast to tumor epithelial cells [35]. This localization has been confirmed by subsequent studies [12], [42]–[44].

In our study, EBER expression was shown by in situ hybridization in a few malignant mammary epithelial cells in 1 out of 10 EBV DNA positive tumors. The absence of signal in 9 out of 10 EBV1 DNA cases could be explained by a lower sensitivity of the in situ hybridization method compared with the PCR method [7], [45]. Moreover, because EBV can be found in different latent phases according to the origin of the cancer cells and the status of the infected cells, unlike the case of other EBV-induced cancers, it is possible that EBER may be poorly expressed or not expressed in all tumor cells in BC [46].

Co-infection with EBV and HPV was detected in only 3 cases (2.4%), which is similar to that observed in BC from Chile (2.1%) [45]. The role of EBV/HPV co-infection in BC is still debated. Recently, it has been postulated that EBV may not be oncogenic when present alone in normal breast tissue, but may gain oncogenic activities in collaboration with other viruses [12]. Moreover it seems possible that EBV-induced carcinogenesis can be favored by a chronic inflammatory status [41]. We could then speculate that HPV infected cells, by releasing IL10 or other Th2 inflammatory cytokine, could create a microenvironment favorable to EBV infection [47]. Interestingly the only case in which we could detect EBER expression was co-infected with HPV. This event could be explained by a higher EBV viral load in this sample, due to HPV co-infection.

Our results suggest an association between the presence of viral DNA and aggressive BC phenotypes (IBC, triple-negative). This corroborates the results of Mazouni et al. [38], who found a higher prevalence of EBV in aggressive ER-negative tumors. It is also consistent with the trend reported by Glenn et al. for EBV, HPV, and MMTV positivity to be associated with higher-grade tumors and a younger age of onset, as well as the correlation reported by Ford et al. between MMTV-LS and high grade of BC [48]. An additional argument is the fact that in Africa and other developing countries, where viruses are expected to play a more important role, BC shows a clear trend toward more aggressive phenotypes and younger age of onset (after correction for younger age of the underlying population) [19], [20].

## Conclusions

Our results are very preliminary; however, they underline the importance of focusing on subgroups, notably aggressive ones, when studying viral etiology in BC. They also highlight the fact that viruses should not be studied in isolation because some kind of viral collaboration may take place. These two recommendations imply that studies about viral etiology in BC should be conducted in much larger samples than has generally been done to date.

Our results support the possible involvement of EBV and, to a lesser extent, HPV in some subgroups of BC. The fact that an EBV vaccine is under development [49], [50] and that an HPV vaccine is already commercially available are strong arguments in support of more research on viral etiology of BC.
